# Societal costs of permanent childhood hearing loss at teen age: a cross-sectional cohort follow-up study of universal newborn hearing screening

**DOI:** 10.1136/bmjpo-2017-000228

**Published:** 2018-02-24

**Authors:** Maria Chorozoglou, Merle Mahon, Hannah Pimperton, Sarah Worsfold, Colin R Kennedy

**Affiliations:** 1 Faculty of Medicine, University of Southampton, Southampton, UK; 2 Language and Cognition Research Department, University College London, London, UK; 3 University Child Health, University Hospital Southampton NHS Foundation Trust, Southampton, UK

**Keywords:** health economics, audiology, deafness, outcomes research, screening

## Abstract

**Objective:**

To investigate the effects in adolescence of bilateral permanent childhood hearing loss (PCHL) > 40 dB and of exposure to universal newborn hearing screening (UNHS) on societal costs accrued over the preceding 12 months.

**Design, setting, participants:**

An observational cohort study of a sample of 110 adolescents aged 13–20 years, 73 with PCHL and 37 in a normally hearing comparison group (HCG) closely similar in respect of place and date of birth to those with PCHL, drawn from a 1992–1997 cohort of 157 000 births in Southern England, half of whom had been exposed to a UNHS programme.

**Intervention:**

Birth in periods with and without UNHS.

**Outcome measures:**

Resource use and costs in the preceding 12-month period, estimated from interview at a mean age of 16.9 years and review of medical records. Effects on costs were examined in regression models.

**Results:**

Mean total costs for participants with PCHL and the HCG were £15 914 and £5883, respectively (difference £10 031, 95% CI £6460 to £13 603), primarily driven by a difference in educational costs. Compared with the HCG, additional mean costs associated with PCHL of moderate, severe and profound severity were £5916, £6605 and £18 437, respectively. The presence of PCHL and an additional medical condition (AMC) increased costs by £15 385 (95% CI £8532 to £22 238). An increase of one unit in receptive language z-score was associated with £1616 (95% CI £842 to £2389) lower costs. Birth during periods of UNHS was not associated with significantly lower overall costs (difference £3594, 95% CI −£2918 to £10 106).

**Conclusions:**

The societal cost of PCHL was greater with more severe losses and in the presence of AMC and was lower in children with superior language scores. There was no statistically significant reduction in costs associated with birth in periods with UNHS.

**Trial registration number:**

ISRCTN03307358, pre-results.

**What is already known on this topic?**

- The consequences of permanent childhood hearing loss (PCHL) can include impairment in language skills and academic achievement which become more marked with more severe PCHL.
- Birth during periods with universal newborn hearing screening (UNHS) is associated with benefits to language and reading abilities.
- The societal costs of prelingual PCHL at 7–9 years increase with its severity and are inversely related to language abilities.

**What this study hopes to add?**

- Total annual costs in adolescents with bilateral PCHL > 40 dB are, on average, 2.7 fold higher than in those with normal hearing.
- PCHL plus specified additional medical conditions is associated with a doubling of annual cost in adolescence compared to that of PCHL alone.
- In adolescents with PCHL, superior language skills are associated with significantly lower societal costs in adolescence but birth in periods with UNHS is not.

## Introduction

Permanent childhood hearing loss (PCHL) is the most common sensory impairment. It is affecting more than 112 per 100 000 children at birth^1^ and incurs substantial economic costs to society,^2^ including those related to special education,^3 4^ employment,^3 4^ vocational rehabilitation,^3 5^ hearing aids, cochlear implants and other medical interventions.^3 5^ These costs to society are particularly high in severe and profound PCHL of prelingual onset and early intervention might confer substantial lifetime financial gains.^5^ Universal newborn hearing screening (UNHS) has been shown to increase the proportion of cases of PCHL that are detected early.^1 6–8^

UNHS adds to financial costs in the first year of life, both because of the cost of administering a UNHS programme, estimated in 1998 as £13 881 per annum for a district with 1000 births,^9^ and because of the additional costs of management of identified cases during the months that would otherwise precede identification of the permanent childhood hearing impairment (PCHI). On the other hand, earlier identification of children born with PCHL can facilitate earlier access to linguistic input and better language and literacy skills^10–16^ and may thus reduce cost subsequent to infancy. More research into the long-term cost-effectiveness of UNHS is needed^17^ and rigorous data on long-term economic consequences of PCHL are required to conduct cost-effectiveness evaluation of UNHS programmes that take into account the long-term consequences of hearing loss.^18^ There is, however, very little direct evidence regarding the long-term economic implications of PCHL^18^ or the effect on them of UNHS for PCHL.

Prior to 2000, attempts to model the long-term costs and outcomes of PCHL were limited by lack of data^3^ and uncertainty regarding the effectiveness of UNHS.^5 19^ From 2003 onwards, UNHS has been implemented in the UK, USA and numerous other countries in the light of high-grade evidence of the benefits of UNHS^14 20^ and there has been significant progress in the provision of paediatric audiological services.^21 22^ In 2009, an estimated 5073 cases of PCHL were detected by UNHS^23^ and accounted for over 43% of the confirmed cases of all 29 medical conditions for which universal newborn screening is mandated in the USA.^23^ The hypothesis that early detection of PCHL reduces the costs of education in the long term^3 12 21 24^ and thus offsets the initial costs of UNHS incurred in infancy warrants examination.

We have previously reported that the economic costs of bilateral PCHL in the preceding year of life among participants in the present study when they were aged 5–10 years were £14 092 for children with PCHL compared with £4207 for normally hearing children. Furthermore, each unit increase in the z score for receptive language among children with PCHL was associated with a statistically significant £2553 reduction in cost in the preceding year. Compared with birth during periods without UNHS, birth during periods with UNHS was associated with a smaller cost reduction of £2213, which fell short of statistical significance.^25^ The participants in that study were subsequently further evaluated at ages 13–20 years in the Hearing Outcomes at Teen Age (HOT) project. We report here on the effects of the severity of their PCHL, of birth during periods with UNHS, of early confirmation of PCHL and of their language ability and reading skills on the societal costs of PCHL.

## Participants and methods

### Study sample

The study sample was drawn from 157 000 children born in two birth cohorts in eight districts of Southern England between 1992 and 1997. The Wessex cohort was born over a 36-month period in four districts that formed the population for the Wessex Trial; a quasiexperimental trial in which UNHS was or was not undertaken in alternating 4–6 month periods in two pairs of hospitals, with UNHS equipment and personnel moving back and forth between the paired hospitals. UNHS increased the rate of early identification for infants with PCHL.^1 6 14^ The Greater London birth cohort was born in two pairs of health districts in Greater London over a 60-month period. Each pair included one of the only two districts in the UK offering UNHS at that time and an immediately neighbouring district.

The language, reading, behaviour and resource use in children with PCHL in these two birth cohorts, and in a normally hearing comparison group (HCG) was assessed in 183 children (120 with PCHL and 63 in the HCG) at a mean age of 7.9 years.^13 15 25 26^ Further assessment of 114 (73 PCHL and 37 HCG) of the sample was undertaken in the Hearing Outcomes at Teen Age (HOT) project at a mean age of 16.9 years.^27–29^ A flow diagram of participants through completion of the HOT study was published in our report of the effect of UNHS on reading comprehension, the primary outcome.^28^ The design included one participant in a normal HCG for every two participants with PCHL in the expectation of providing three equally sized groups: participants with PCHL exposed and not exposed to a UNHS programme and participants in the HCG.

Written informed consent was obtained from principal caregivers and the teenage participants.

### Measures

Severity of PCHL was classified according to average pure tone thresholds across four frequencies of sound in the better ear as moderate (> 40–70 decibels (dB) hearing level), severe (71–95 dB) or profound (>95 dB). Intellectual disability (defined by non-verbal ability scores), genetic syndrome, visual loss and cerebral palsy were recorded as additional medical conditions (AMC). Methods of assessing participants’ reading comprehension, receptive language ability, non-verbal ability and other outcomes have been reported previously.^27–29^ Occupation of the head of the household and maternal educational level were, as in our previous 2001–2004 assessment,^25^ described using UK 2001 national census categories.^30^

### Resource use and costs

Resource use was considered from the healthcare provider, National Health Service (NHS), Personal Social Services (PSS) and societal perspective, including costs borne by the family. It was estimated by retrospective examination of each child’s audiology records coupled with data on resource use in seven domains (*vide infra*) obtained by the four study research assistants at interviews of parents in their homes using instruments previously developed for our 2001–2004 study of the same families.^25^ These data covered use of a range of services during the preceding 6 months, a period short enough for recall to be reliable, and were extrapolated to provide an estimate of annual cost. The research staff involved in the follow-up study were unaware of the age of initial referral and management and, in the case of the Wessex subgroup, blind to whether or not the child was born in a period with UNHS.

All unit costs adopted in the analysis were based on 2012/2013 price indexes. Health and Community Health Services pay and price indices were used to inflate costs, where appropriate.^31^ Published sources of unit costs included NHS Reference Costs^32^ and the PSS Resource Unit estimates.^31^ Unit costs for schooling were accessed individually for each school (from the UK Department for Education for state schools and from individual schools for the private sector) and the mean unit cost estimates for each type of school were included in the analysis.^33^ Other unit cost estimates were obtained from local authorities and local suppliers.

Costs, estimated at the individual person level, are presented in the form of group means and SDs in seven domains: hospital outpatient and inpatient services, including cochlear implantation; community health and social care services; respite and foster care; local authority loaned/provided equipment and home adaptations; educational services including special educational needs provision; parents’ lost productivity; and other household-borne costs, including household purchased equipment and home adaptations.

### Statistical analysis

The primary outcome variable for the economic study reported here was total costs which were compared between the teenagers with PCHL and the HCG. As the time frame for the cost analysis was 1 year, discounting applied to economic evaluations in excess of a 1-year time frame was not necessary.

The target sample size of 96 children with PCHL for the HOT project, that is, 80% of the participants with PCHL that had been assessed at a mean age of 7.9 years, was estimated to provide 90% power at 5% significance level (two tailed) to detect a 0.67 SD effect size of UNHS on reading comprehension, the prespecified primary outcome measure in the HOT study, in participants with PCHL. Sample size was determined by the above power calculation rather than any separate power calculation relating to power to detect group differences in costs, the secondary outcome reported here.

Among participants with PCHL, the effect on costs was assessed in four regression models, each with one independent variable of interest: birth during periods with UNHS; ‘early’ confirmation of PCHL; receptive language ability z-score; and reading comprehension z-score. These effects are presented unadjusted and adjusted in two regression models. The first model adjusted for cochlear implantation and the presence of AMCs and the second model added severity of PCHI into that regression model.

As violation of normality was confirmed (Shapiro-Wilk P<0.05), mean differences between groups are presented with 95% CIs estimated by bootstrapping (1000 replications). In addition to conventional ordinary least squares (OLS) regression analysis, generalised linear models (GLM) using non-normal distributions, and the alternative model specifications were examined for robustness to deviations from normality and equality of variance in costs.^34^ OLS and GLM analyses gave very similar results so we used OLS findings with robust SEs. All analyses were carried out using STATA V.12 and R V.3.1.1.^35–37^

## Results

Four of 114 participants in the HOT study did not return the completed economic questionnaire and resource use is therefore reported in 110 participants in this economic study. The mean (SD) age of the participants was 16.9 (1.4) years. Of the participants with PCHL, 32 (44%), 18 (25%) and 23 (32%) had moderate, severe and profound PCHL, respectively (table 1). For the PCHL group there were no significant differences of gender, severity of PCHL, mother’s educational qualifications, or English as the main language at home between participants and those lost to follow-up in the larger sample of 120 children with PCHL, who had been assessed at 7.9 years.^27^ Additional demographic characteristics by UNHS status and by timing of confirmation of PCHL are presented in online supplementary appendix table 1. Online supplementary appendix table 1 and our previous reports indicate that the (approximately) half of our study population with PCHL that was born in periods with UNHS was similar to the other half born in periods without UNHS with respect to the severity of their PCHL. That is to say severity of PCHL was not a confounder of UNHS status when considering the effect of UNHS on costs. Resource use (table 2) was combined with unit costs (table 3) to derive total costs in all participants (table 4).

10.1136/bmjpo-2017-000228.supp1 Supplementary file 1

**Table 1:** Sociodemographic and clinical characteristics of study participants

Variable	Bilateral permanent childhood hearing loss >40 dB				HCG n=37
	Moderate n=32	Severe n=18	Profound n=23	Total n=73	
Age mean (SD) in years	16.9 (1.4)	17.5 (1.4)	16.8 (1.5)	17.0 (1.4)	16.3 (1.2)
Female, n (%)	16 (50)	9 (50)	10 (44)	35 (48)	13 (35)
Mode of communication, n (%)					
Oral	22 (69)	10 (56)*	11 (48)*	43 (59)	–
Sign	0	0	1 (4)	1 (1)	–
More than one mode	10 (31)	8 (44)	11 (48)	29 (40)	–
UNHS status, n (%)					
Born in periods without UNHS	14 (44)	10 (56)	14 (61)	38 (52)	–
Age PCHL confirmed, n (%)					
>9 completed months	16 (50)	12 (67)	11 (48)	39 (53)	–
English main language at home, n (%)	31 (97)	15 (83)	18 (78)	64 (88)	–
Mother’s educational qualifications†, n (%)					
No qualifications	3 (9)	2 (11)	2 (9)	7 (10)	2 (5)
<5 O-level examinations	4 (12)	1 (6)	4 (17)	9 (12)	3 (8)
≥5 O-level examinations	9 (28)	7 (39)	8 (35)	24 (33)	13 (35)
Some A-level examinations	8 (25)	4 (22)	2 (9)	14 (19)	1 (3)
≥University degree	8 (25)	4 (22)	7 (30)	19 (26)	18 (49)
Social class‡, n (%)					
Higher occupations	15 (47)	10 (56)	11 (48)	36 (49)	26 (70)
Intermediate occupations	10 (31)	3 (17)	5 (22)	18 (25)	8 (22)
Lower occupations	4 (12)	0	5 (22)	9 (12)	3 (8)
Never worked and long term unemployed	3 (9)	5 (28)	2 (9)	10 (14)	0
Family income, n (%)					
<10 000	4 (13)	2 (12)	0	6 (9)	0
10 000–20 000	6 (20)	2 (12)	7 (33)	15 (22)	4 (11)
21 000–30 000	2 (7)	4 (24)	2 (10)	8 (12)	7 (19)
31 000–40 000	7 (23)	2 (12)	4 (19)	13 (19)	4 (11)
41 000–50 000	3 (10)	2 (12)	1 (5)	6 (9)	5 (14)
>50 000	8 (27)	5 (29)	7 (33)	20 (29)	17 (46)
Additional medical conditions§, n (%)	9 (28)	3 (11)	4 (17)	16 (22)	1 (3)
Hearing aids, n (%)					
No aid	3 (9)	2 (17)	11 (48)	16 (22)	–
One aid	4 (12)	1 (6)	3 (13)	8 (11)	–
Two aids	25 (78)	15 (83)	9 (39)	49 (67)	–
Number of cochlear implant(s), n (%)					
None	32 (100)	17 (94)	11 (48)	59 (81)	–
1	–	1 (6)	7 (30)	9 (12)	–
2	–	–	5 (22)	5 (7)	–

*Language other than English in one participant.

†O level refers to ‘ordinary levels’ UK qualification achieved at 16 years. A level refers to ‘advanced levels’ UK qualification achieved at 18 years.

‡Classified according to UK National Census 2002.

§These were severe visual impairment, cerebral palsy, mental retardation and genetic syndrome.

HCG, hearing comparison group; PCHL, permanent childhood hearing loss >40 dB; UNHS, universal newborn hearing screening.

**Table 2:** Estimated group mean resource use in preceding 12 months

Resource items*	Bilateral permanent childhood hearing loss >40 dB				HCG (n=37)
	Moderate (n=32)	Severe (n=18)	Profound (n=23)	Total (n=73)	
	Mean (SD)	Mean (SD)	Mean (SD)	Mean (SD)	Mean (SD)
Community and social care services contacts					
General practitioner	2.25 (3.04)	2.89 (3.51)	2.70 (2.80)	2.55 (3.06)	1.46 (2.14)
Practice nurse	0.56 (1.37)	0.44 (1.10)	16.52 (74.90)	5.56 (42.09)	0.16 (0.55)
Community nurse	–	0.44 (1.89)	0.26 (1.25)	0.19 (1.16)	0.05 (0.33)
Community paediatrician	0.13 (0.49)	0.11 (0.47)	0.09 (0.42)	0.11 (0.46)	0.11 (0.66)
Dentists	1.44 (1.27)	1.67 (1.41)	1.39 (1.12)	1.48 (1.25)	1.35 (1.16)
Orthodontist	0.88 (2.15)	0.67 (1.94)	1.57 (2.95)	1.04 (2.38)	1.51 (2.77)
Optician	0.63 (0.94)	0.78 (1.00)	1.22 (1.00)	0.85 (1.00)	0.65 (0.95)
Chiropodist	0.44 (1.50)	–	–	0.19 (1.01)	–
Physiotherapist	0.63 (1.86)	0.22 (0.65)	2.35 (10.03)	1.07 (5.76)	0.38 (1.62)
Speech and language	1.94 (9.25)	17.56 (56.85)	18.35 (38.43)	10.96 (36.27)	0.05 (0.33)
Health visitor	1.44 (1.70)	1.11 (1.23)	1.30 (1.66)	1.32 (1.57)	–
Home visitor	0.31 (1.15)	0.44 (1.46)	0.96 (2.75)	0.55 (1.86)	–
Social worker	–	5.33 (15.52)	–	1.32(7.89)	–
Counsellor	0.81 (4.25)	–	0.61 (2.04)	0.55 (3.02)	0.05 (0.33)
Community psychologist	–	–	–	–	0.11 (0.46)
Community psychiatrist	–	0.11 (0.47)	0.09 (0.42)	0.05 (0.33)	0.05 (0.33)
Osteopath	–	–	–	–	–
Audiologist	0.44 (0.84)	0.44 (1.10)	0.17 (0.58)	0.36 (0.84)	–
Other	0.06 (0.25)	0.11 (0.47)	–	0.05 (0.28)	–
Other care service					
Respite care (days)	0.38 (2.12)	4.00 (12.35)	4.13 (13.20)	2.45 (9.70)	–
Foster care (days)	6.66 (37.70)	–	–	2.92 (24.90)	–
Hospital outpatient, attendances					
Category 1 (ENT)	0.19 (0.78)	0.44 (1.46)	0.09 (0.42)	0.22 (0.92)	0.22 (1.03)
Category 2 (A&E)	0.81 (2.02)	0.56 (1.50)	0.87 (2.40)	0.77 (2.02)	0.86 (1.86)
Category 3 (other)	0.19 (0.78)	0.11 (0.47)	0.26 (1.25)	0.19 (0.89)	0.22 (0.79)
Hospital inpatient admissions (days)					
Cochlear implant	–	–	0.17 (0.39)	0.05 (0.23)	–
Total days	0.22 (0.94)	–	0.17 (0.49)	0.15 (0.68)	–
Education, number of children attending: n (%)					
Mainstream school	19 (65.5)	5 (35.7)	4 (17.4)	28 (42.4)	33 (100.0)
Mainstream school with unit for deaf	3 (10.3)	5 (35.7)	5 (21.7)	13 (19.7)	–
Special school for deaf	1 (3.5)	2 (14.3)	11 (47.8)	14 (21.2)	–
Other special school	5 (17.2)	2 (14.3)	2 (8.7)	9 (13.6)	–
Other school	1 (3.5)	–	1 (4.4)	2 (3.0)	–
Residential school	1 (3.4)	2 (14.3)	10 (43.5)	13 (19.7)	–

*Medication costs are not included. Thirty-four of 110 reported having used medication which was unnamed in 16. Dose and frequency information was seldom available.

A&E, Accident and Emergency; ENT, Ear, Nose and Throat; HCG, hearing comparison group.

**Table 3:** Unit costs of resource items

Resource items	Unit cost or range*	Source of unit cost
Community and social care services, per contact hour		
Practice nurse	41.0 (35.0–53.0)	Curtis^31^
Community nurse	39.0 (33.0–43.0)	Curtis^31^
Community paediatrician	223.0	NHS Reference Costs^32^
Dentists	115.0	NHS Reference Costs^32^
Orthodontist	45.0	NHS Reference Costs^32^
Optician	138.0	NHS Reference Costs^32^
Chiropodist	41.0 (33.0–45.0)	Curtis^31^
Physiotherapist	47.0 (37.0–53.0)	Curtis^31^
Speech and language	74.0 (52.0–87.0)	Curtis^31^
Health visitor/research therapist	44.0 (33.0–54.0)	Curtis^31^
Social worker	54.0 (34.0–150.0)	Curtis^31^
Counsellor	35.6–90.1	Inflated PSSRU, 2007^34^
Community psychologist	60.0–136.0	Curtis^31^
Community psychiatrist	60.0	Curtis^31^
Osteopath	35.0–50.0	NHS Reference Costs^32^
Audiologist	150.0	NHS Reference Costs^32^
General practitioner, per consultation	53.0 (43.0–63.0)	Curtis^31^
Other care service, per week		
Residential respite care	268.0 (71.0–413.0)	Inflated PSSRU, 2011^35^
Foster care	637.0	Inflated PSSRU, 2011^34^
Hospital outpatient, per attendance†		
Category 1 (ENT)	71.7 (45.0–98.0)	NHS Reference Costs^32^
Category 2 (A&E)	137.6 (106.0–197.0)	NHS Reference Costs^32^
Category 3 (other)	268.6 (205.0–351.0)	NHS Reference Costs^32^
Hospital inpatient admissions, per admission		
Cochlear implant‡	20 333.0–30 709.0	NHS Reference Costs^32^
Paediatric ward	757.0–12 281.0	NHS Reference Costs^32^
Other	545.0–1846.0	NHS Reference Costs^32^
Education, per year		
Mainstream school	4581.0	Department of Education, 2012^36^
Mainstream school with special unit	4819.0	Department of Education, 2012^36^
Special school for the physically disabled	17 795.0–27 000.0	Local authority (Southampton)
Residential school	61 859.0–167 268.0	NASS^33^ and individual schools
Special school for learning difficulties/deaf	15 580.0–25 833.0	Local authority (Southampton)
Equipment loaned, per year		
Digital hearing aid	126	NHS Reference Costs^32^
Wheelchair	172.0	Inflated PSSRU, 2011
Loop system	137.0–1200.0	Local provider
Vibrating alarm clock	15.0–85.0	Local provider
Doorbell/light	8.5–59.9	Local provider
Fire alarm and flashing lights	7.7–138.0	Local provider
Light-up phone	34.8–70.8	Local provider
Local authority provided home adaptations, unit cost		
Bathing equipment	4539	Local authority (Southampton)
Adapted shower	5000	Local authority (Southampton)
Accessible kitchen built	483	Curtis^31^

Values are £2013.

*Ranges of unit costs are specified where unit costs varied according to location or intensity of care provided.

†Hospital outpatient attendances are categorised as low, medium and high cost services.

‡Includes cost of cochlear implant equipment and surgical procedure and other inpatient costs.

A&E, Accident and Emergency; ENT, Ear, Nose and Throat; NASS, National Association of Independent Schools and Non-Maintained Special Schools; NHS, National Health Service; PSSRU, Personal Social Services Resource Unit.

**Table 4:** Annual mean costs by cost category for health and social service use and UNHS status

Cost domain	Children with PCHL				HCG (n=37) Mean (SD)	PCHL versus HCG Mean difference (Bootstrap SE)	95% CI
	Moderate (n=32)	Severe (n=18)	Profound (n=23)	All PCHL (n=73)			
	Mean (SD)	Mean (SD)	Mean (SD)	Mean (SD)			
Hospital outpatient	168.94 (330.48)	125.11 (274.74)	202.96 (693.85)	168.85 (461.30)	141.19 (307.21)	27.66 (76.15)	−121.60 to 176.92
No UNHS	238.57 (383.15)	85.00 (191.79)	60.00 (224.50)	132.37 (291.70)			
UNHS	114.78 (282.50)	175.25 (361.72)	425.33 (1072.67)	208.46 (595.76)			
Hospital inpatient	165.59 (712.58)	–	65.83 (218.09)	93.33 (487.75)	–	93.33 (60.86)	−25.96 to 212.61
No UNHS	108.14 (404.63)		54.07 (202.32)	59.76 (271.61)			
UNHS	210.28 (892.13)		84.11 (252.33)	129.77 (648.75)			
Cochlear implant	–	–	2652.13 (7001.67)	835.60 (4064.28)	–	835.6 (415.43)	21.37 to 1649.83
No UNHS			2904.71 (7383.66)	1070.16 (4601.24)			
UNHS			2259.22 (6777.67)	580.94 (3436.90)			
Hospital total	400.16 (911.06)	191.78 (353.71)	2947.00 (7170.03)	1151.21 (4195.99)	141.19 (307.21)	1010.02 (578.76)	−124.33 to 144.37
No UNHS	432.43 (529.46)	205.00 (366.22)	3040.21 (7508.91)	1333.34 (4657.81)			
UNHS	375.06 (1139.16)	175.25 (361.72)	2802.00 (7050.75)	953.46 (3687.43)			
Community and social care	776.09 (916.37)	2433.32 (4906.58)	2590.92 (3608.70)	1756.51 (3284.68)	503.79 (437.67)	1252.72 (402.05)	464.71 to 2040.73
No UNHS	930.67 (1185.75)	2799.16 (6461.98)	3061.47 (4450.85)	2207.41 (4312.64)			
UNHS	655.86 (648.94)	1976.01 (2085.33)	1858.94 (1621.98)	1266.97 (1460.70)			
Respite care	100.13 (566.39)	–	174.13 (835.10)	98.75 (596.21)	–	98.75 (51.45)	−2.08 to 199.59
No UNHS	228.86 (856.31)		286.07 (1070.38)	189.71 (820.98)			
UNHS	–		–	–			
Foster care	604.77 (3421.08)	–	–	265.10 (2265.05)	–	265.10 (226.56)	−178.93 to 709.14
No UNHS	–			–			
UNHS	1075.14 (4561.45)			552.93 (3271.18)			
Equipment and home adaptations	129.51 (220.89)	200.33 (238.68)	63.48 (122.87)	126.17 (204.22)	–	126.17 (22.49)	82.10 to 170.24
No UNHS	168.64 (286.39)	223.00 (282.84)	36.43 (72.39)	134.24 (237.31)			
UNHS	99.07 (154.97)	172.00 (183.95)	105.56 (172.43)	117.41 (163.97)			
Educational services	9250.30 (7817.55)	9743.14 (9356.48)	17 752.05 (10 077.91)	12 082.50 (9655.09)	5330.24 (1690.63)	6752.26 (1117.46)	4562.08 to 8942.44
No UNHS	12 020.40 (9894.91)	9940.71 (9096.11)	17 080.49 (10 828.66)	13 429.16 (10 251.11)			
UNHS	7095.77 (5033.60)	9520.87 (10 268.11)	18 796.71 (9313.30)	10 658.89 (8907.32)			
Lost productivity	16.88 (59.48)	–	82.61 (315.73)	33.42 (182.09)	12.97 (78.91)	20.45 (24.39)	−27.35 to 68.25
No UNHS	21.43 (80.18)		107.14 (400.89)	47.37 (246.86)			
UNHS	13.33 (38.81)		44.44 (101.38)	18.29 (58.69)			
Other household	521.25 (2040.87)	461.22 (912.33)	710.09 (1289.46)	565.95 (1583.72)	38.92 (236.73)	527.03 (153.73)	225.72 to 828.34
No UNHS	927.43 (3071.52)	208.60 (390.24)	686.57 (1372.79)	649.53 (2023.91)			
UNHS	205.33 (373.21)	777.00 (1272.98)	746.67 (1227.84)	475.20 (915.15)			
*Total costs excluding education*	2548.77 (4914.31)	3286.65 (5160.29)	6568.22 (8890.53)	3997.12 (6633.79)	696.88 (674.54)	3300.24 (644.21)	2037.62 to 4562.86
No UNHS	2709.45 (4331.12)	3435.76 (6541.88)	7217.90 (10 191.51)	4561.59 (7602.96)			
UNHS	2423.80 (5445.76)	3100.26 (3094.22)	5557.61 (6833.21)	3384.26 (5435.40)			
*Total costs including education*	11 799.07 (10 186.17)	12 488.50 (13 179.43)	24 320.28 (16 381.95)	15 914.10 (14 167.56)	5883.05 (2075.50)	10 031.05 (1822.27)	6459.47 to 13 602.62
No UNHS	14 729.85 (12 598.90)	12 382.40 (13 892.31)	24 298.39 (19 877.75)	17 637.35 (16 401.04)			
UNHS	9519.57 (7432.83)	12 621.12 (13 178.09)	24 354.32 (9794.52)	14 043.15 (11 198.32)			

HCG, hearing comparison group; PCHL, permanent childhood hearing loss > 40 dB; UNHS, universal newborn hearing screening.

### Comparison between those with PCHL and the HCG

The mean (SD) cost estimates for the teenagers with PCHL and the HCG were £15 914 (14 168) and £5883 (2076), respectively (mean difference (95% CI) £10 031 (£6459 to £13 603), P<0.001) (table 4). Both educational costs and the sum of all other costs differed significantly between these groups. The educational cost difference of £6752 was the main cost driver (table 4).

### Comparisons within the PCHL group

#### Effect of severity of PCHL

Moderate, severe and profound PCHL were associated with mean costs of £11 799, £12 489 and £24 320, respectively (table 4). The mean cost differences from the HCG for moderate, severe and profound PCHL were £5916, £6605 and £18 437, respectively. The mean difference (95% CI) in the cost of profound compared with other severities of PCHL was £12 273 (£4808 to £19 738) (P=0.002). The higher cost of attendance at boarding and independent special schools was the main cost driver: the percentage (95% CI) of teenagers with moderate, severe and profound PCHL attending residential schools was 8 (1% to 42%), 15 (4% to 47%) and 77 (46% to 93%), respectively, and the percentage (95% CI) attending mainstream schools was 68 (48% to 83%), 18 (7% to 37%) and 14 (5% to 33%), respectively. Community health, social care and hospital-based service costs all increased significantly with severity (table 4). For those with profound PCHL, the cost of cochlear implantation was only incurred during the assessed period of resource use in a small proportion but the group mean cost of cochlear implant (£2652) was nevertheless a key cost driver (table 4).

#### Effect of AMCs

Of those participants with AMC, 56%, 19% and 25% had moderate, severe, and profound PCHL, respectively. Overall, the presence of a medical condition additional to PCHL was associated with higher mean costs (95% CI) by £15 385 (£8533 to £22 238). This cost difference was £21 876 (£13 024 to £30 728) for loss of vision, £14 200 (£5620 to £22 780) for genetic syndromes, £11 728 (£5456 to £18 000) for intellectual disability and £1642 (−£8013 to £11 296) for cerebral palsy. For the moderate, severe and profound groups, the mean costs by severity for the PCHL group with and without AMC (n=1657) were £22 436, £36 318, £33 990 and £7637, £7723, £22 285, respectively.

#### Effect of birth during periods with UNHS

In children with PCHL, the total mean annual costs associated with birth in periods with and without UNHS were £14 043 and £17 637, respectively (mean difference £3594, 95% CI −£2918 to £10 106, P=0.28) (table 5). The cost difference was mainly associated with placement of a higher percentage (95% CI) of those born in periods with UNHS in local mainstream schools, 61 (41% to 78%) compared with 39 (22% to 59%).

**Table 5:** Total costs in preceding year in relation to presence of UNHS, confirmation by age 9 months, receptive language ability and reading

Variable	Unadjusted costs		Costs adjusted for cochlear implantation and presence of an additional medical condition		Costs adjusted for cochlear implantation, presence of an additional medical condition and severity of PCHL	
	β coefficient (95% CI)	P	β coefficient (95% CI)	P	β coefficient (95% CI)	P
Born during periods with UNHS	−3594.21 (−10 105.9 to 2917.6)	0.28	−970.46 (−6099.42 to 4158.50)	0.71	−228.35 (−5303.44 to 4846.74)	0.93
Confirmation of PCHL at >9 months	−2824.11 (−9381.5 to 3733.3)	0.39	−408.01 (−5662.35 to 4846.32)	0.88	47.99 (−5098.08 to 5194.06)	0.98
Receptive language z-score	−1615.84 (−2389.3 to −842.4)	<0.001	−1011.33 (−2086.17 to 63.50)	0.065	−649.90 (−1745.77 to 445.97)	0.24
Reading comprehension z-score	−1886.94 (−3797.4 to 23.5)	0.05	−669.91 (−2516.30 to 1176.48)	0.47	−328.15 (−2478.69 to 1822.39)	0.76

PCHL, permanent childhood hearing loss > 40 dB hearing level (HL); UNHS, universal newborn hearing screening.

#### Effect of early confirmation

Early confirmation of PCHL, like birth in periods with UNHS, was not associated with a significant difference in cost (cost difference £2824, 95% CI £3733 to £9382, P=0.39) (table 5). The cost difference remained non-significant when only participants without an AMC (n=57) were included in the analysis (difference £1487, 95% CI −£5164 to £8138). As there is an association between early confirmation of PCHL and greater severity of PCHL, it is necessary to adjust for severity in multivariate analysis. In that analysis (right hand model in table 5) no effect of early confirmation of PCHL on costs is apparent.

#### Effects of language and reading z-score

Each unit increase in receptive language ability z-score was associated with significantly lower annual costs by £1616 (95% CI £842 to £2389) (P<0.001). Similarly, each unit increase in reading ability z-score was associated with marginally significantly lower annual costs by £1887 (95% CI −£1234 to £3516, P=0.053) (table 5). Both of these effects fell short of statistical significance in multivariate analysis, although the effect of receptive language score on costs remained marginally significant (0.05<P<0.1) after adjusting for the effects of cochlear implantation and AMC on costs but not when additional adjustment for severity of PCHL was added into the model (table 5). This is considered further in the Discussion section.

## Discussion

Compared with participants of similar age with normal hearing, the presence of bilateral PCHL > 40 dB at ages 13–20 years was associated with 2.7-fold higher costs in the preceding 12-month period and the presence of prespecified medical conditions in addition to PCHL increased costs almost twofold. No statistically significant association was found between either birth in periods with UNHS or early confirmation of PCHL and costs in adolescence.

In participants with PCHL, superior language skills were associated with lower costs and this remained marginally significant (0.05<P<0.1) after adjustment for the presence of a cochlear implant or an AMC but not after adjustment for severity of PCHL. Superior receptive language scores were associated with significantly lower costs in the same birth cohort when assessed 9 years earlier at ages 5–10 years.^25^ The costs associated with superior language at ages 5–10 years remained significantly lower after adjustment for severity of PCHL (ref 25, table 5) but not in the present study at ages 13–20 years. However, the latter regression model may represent overadjustment if the effect of severity of PCHL on cost is mediated by the well-recognised inverse relationship between severity of PCHL and receptive language,^2–5^ as seems particularly likely in the case of educational costs. In that earlier report, the reduction in cost associated with a unit increase in the z-score for receptive language was equivalent to 28.6% of total excess group mean annual costs associated with PCHL, whereas in the present study it was shown that the equivalent figure had fallen to 16.1% of those costs in adolescence. The absence of significant difference in the current evaluation may be due to this lower contribution of receptive language to total excess annual cost in adolescence compared with children 5–10 years old, but the total overall costs (from 0 to 20 years) may still be affected by receptive language score and further economic evaluations are required to assess this. Taken together, the previous and present evaluations in our study cohort do provide some support for the hypothesis that superior language scores are associated with lower costs in PCHL during childhood and adolescence. A full economic evaluation integrating all costs from birth to adolescence and the costs of screening would be required to better assess the cost-effectiveness of universal hearing screening.

The 7-year range of age at time of assessment is consequent on the study design, that is, an evaluation of a 5-year birth cohort conducted over a 3-year study period. This is both a limitation, in that it makes the estimate for any 1 year of age less precise, and a strength, in that it makes the findings more generalisable to teenagers in general.

The two principal limitations of the study were the modest study size with 120 participants at the outset and further reduction in its power to look at subgroups (eg, severities of PCHL) by slow but steady loss of participants over the 17 years of follow-up, although without apparent attrition bias.^27 28^ This long period of follow-up provides data that are rare because of the extreme difficulty of obtaining them and also limits the generalisability of these findings to babies currently being born because newborns will now be offered paediatric audiology services that have adapted to UNHS in the 25 years since recruitment of newborns into our study began.

The receptive language skills, which we found to be superior in children with PCHL born in periods with UNHS in our study population,^13^ should receive greater benefit in current and future birth cohorts than those observed in our study cohort because of the much clearer care pathways that now lead from UNHS to early intervention for PCHL. We therefore predict that the lower costs associated with superior language skills that we observed in this study population in childhood and adolescence will be more strongly associated with UNHS in a current or future birth cohort. In other words, management strategies made possible by UNHS could have the potential to lead to significantly reduced future costs as a result of superior language skills.

Reports of societal costs in more recent and larger birth cohorts exposed to UNHS, such as those reported in the Netherlands^8^ and Australia,^38^ are therefore awaited to confirm and extend our observations. Future research should, in addition, consider extracting resource utilisation from large national databases as a cost-effective approach to economic evaluation of UNHS.

The messages for policymakers of the associations observed, both at 5–10 years and in adolescence, in the birth cohort reported here include confirmation of the association between PCHL and significantly increased cost to society and the suggestion that interventions that improve language skills may bring benefit to the individual with PCHL and may be seen as a financial investment that should bring longer term cost savings through reductions in educational spending. These findings need confirmation in other larger birth cohorts.
