# Supplementary material for: Societal costs of permanent childhood hearing loss at teen age: a cross-sectional cohort follow-up study of universal newborn hearing screening
Source: BMJ Paediatr Open. 2018 Feb 24;2(1):e000228. doi: 10.1136/bmjpo-2017-000228 (PMC5887866; doi:10.1136/bmjpo-2017-000228)
Supplement: Supplementary file 1 [file bmjpo-2017-000228supp001.pdf]

Appendix Table 1 Sociodemographic and Clinical Characteristics of PCHL

Variable		UNHS status		Age of confirmation of PCHL	
		Not screened (n=38)	Screened (n=35)	≤ 9 months (n=34)	> 9 months (n=39)
	Mean age (SD) in years	17.3 (1)	16.7 (2)	16.7 (2)	17.3 (1)
		**n (%)**	**n (%)**	**n (%)**	**n (%)**
	Female	19 (50)	16 (46)	16 (47)	19 (49)
**Mode of communication**	English	18 (47)	23 (66)	21 (62)	20 (51)
	Spoken language not English	1 (3)	1 (3)	0	2 (5)
	Nonverbal gesture	0	1 (3)	1 (3)	0
	More than one mode	19 (50)	10 (29)	12 (35)	17 (44)
**English main language at home**		30 (79)	34 (97)	33 (97)	31 (80)
**Mother's educational qualifications**					
	No qualifications	4 (10)	3 (9)	2 (6)	5 (13)
	<5 O-level examinations	2 (5)	7 (20)	6 (18)	3 (8)
	≥5 O-level examinations	12 (32)	12 (34)	12 (35)	12 (31)
	Some A-level examinations	7 (18)	7 (20)	5 (15)	9 (23)
	≥University degree	13 (34)	6 (17)	9 (26)	10 (26)
**Social class**	Higher occupations	21 (55)	15 (43)	18 (53)	18 (46)
	Intermediate occupations	9 (24)	9 (26)	9 (26)	9 (23)
	Lower occupations	4 (10)	5 (14)	5 (15)	4 (10)
	Never worked and LT unemployment	4 (10)	6 (17)	2 (6)	8 (20)
**Severity of Loss**	Moderate	14 (37)	18 (51)	16 (47)	16 (41)
	Severe	10 (26)	8 (23)	6 (18)	12 (31)
	Profound	14 (37)	9 (26)	12 (35)	11 (28)
**Family Income**	<10k	0	6 (17)	1 (3)	5 (13)
	10-20k	10 (29)	5 (14)	8 (23)	7 (18)
	21-30k	6 (16)	2 (6)	4 (12)	4 (10)
	31-40k	7 (18)	6 (17)	5 (15)	8 (20)
	41-50k	2 (5)	4 (11)	5 (15)	1 (3)
	>50k	9 (24)	11 (31)	10 (29)	10 (26)
	Missing	4 (10)	1 (3)	1 (3)	4 (10)
**Additional Medical Conditions**		7 (18)	5 (14)	7 (21)	5 (13)
**Hearing Aids**	No aid	6 (16)	10 (29)	10 (29)	6 (15)
	One aid	4 (10)	4 (11)	3 (8.8)	5 (13)
	Two aids	28 (74)	21 (60)	21 (62)	28 (72)
**Number of Cochlear Implant(s)**					
	None	30 (79)	29 (83)	27 (79)	32 (82)
	One implant	4 (10)	5 (14)	5 (15)	4 (10)
	Two implants	4 (10.5)	1 (2.9)	2 (5.9)	3 (7.7)

PCHL= Bilateral permanent childhood hearing loss ≥40 dB

Appendix Table 1 Annual mean costs by cost category and early confirmation of PCHL

Cost Domain	Children With PCHL								HCG (n=37)		PCHL vs HCG	95% Confidence interval
	Moderate (n=32)		Severe (n=18)		Profound (n=23)		All PCHL (n=73)				Mean Diff	
	Mean	(SD)	Mean	(SD)	Mean	(SD)	Mean	(SD)	Mean	(SD)	(Bootstrap SE)	
**Hospital outpatient**	**168.94**	**(330.48)**	**125.11**	**(274.74)**	**202.96**	**(693.85)**	**168.85**	**(461.30)**	**141.19**	**(307.21)**	**27.66 (76.15)**	**-121.60 to 176.92**
≤ 9 months	245.25	(435.95)	162.00	(256.53)	319.00	(934.78)	256.59	(625.09)				
> 9 months	92.63	(152.46)	106.67	(292.62)	76.36	(253.27)	92.36	(225.79)				
**Hospital inpatient**	**165.59**	**(712.58)**	-		**65.83**	**(218.09)**	**93.33**	**(487.75)**	-		**93.33 (60.86)**	**-25.96 to 212.61**
≤ 9 months	236.56	(946.25)			63.08	(218.53)	133.59	(658.11)				
> 9 months	94.63	(378.50)			68.82	(228.24)	58.23	(268.18)				
**Cochlear Implant**	-		-		**2,652.13**	**(7,001.67)**	**835.60**	**(4,064.28)**	-		**835.6 (415.43)**	**21.37 to 1649.83**
≤ 9 months					1,694.42	(5,869.63)	598.03	(3,487.08)				
> 9 months					3,696.91	(8,225.10)	1,042.72	(4,543.53)				
**Total Hospital care**	**400.16**	**(911.06)**	**191.78**	**(353.71)**	**2,947.00**	**(7,170.03)**	**1,151.21**	**(4,195.99)**	**141.19**	**(307.21)**	**1010.02 (578.76)**	**-124.33 to 2144.37**
≤ 9 months	481.81	(1,233.63)	262.00	(473.00)	2,101.50	(6,144.99)	1,014.68	(3,739.36)				
> 9 months	318.50	(423.46)	156.67	(296.01)	3,869.36	(8,352.83)	1,270.23	(4,602.73)				
**Community & Social Care**	**776.09**	**(916.37)**	**2,433.32**	**(4,906.58)**	**2,590.92**	**(3,608.70)**	**1,756.51**	**(3,284.68)**	**503.79**	**(437.67)**	**1,252.72 (402.05)**	**464.71 to 2040.73**
≤ 9 months	709.33	(684.54)	4,671.10	(8,160.16)	1,573.97	(1,488.37)	1,713.63	(3,623.11)				
> 9 months	842.85	(1,121.32)	1,314.42	(1,685.67)	3,700.32	(4,859.87)	1,793.90	(3,006.61)				
**Respite Care**	**100.13**	**(566.39)**	-		**174.13**	**(835.10)**	**98.75**	**(596.21)**	-		**98.75 (51.45)**	**-2.08 199.59**
≤ 9 months	200.25	(801.00)					94.24	(549.48)				
> 9 months	-				364.09	(1,207.55)	102.69	(641.31)				
**Foster Care**	**604.77**	**(3,421.08)**	-		-		**265.10**	**(2,265.05)**	-		**265.10 (226.56)**	**- 178.93 709.14**
≤ 9 months	1,209.54	(4,838.14)					569.19	(3,318.94)				
> 9 months	-						-					

Appendix Table 2 Annual mean costs by cost category and early confirmation of PCHL (concl)

Cost Domain	Children With PCHL								HCG (n=37)		Mean Diff PCHL vs HCG	95% Confidence interval	
	Moderate (n=32)		Severe (n=18)		Profound (n=23)		All PCHL (n=73)						
	Mean	(SD)	Mean	(SD)	Mean	(SD)	Mean	(SD)	Mean	(SD)	(Bootstrap SE)		
**Equipment & home adaptations**	**129.51**	**(220.89)**	**200.33**	**(238.68)**	**63.48**	**(122.87)**	**126.17**	**(204.22)**	**-**		**126.17 (22.49)**	**82.10**	**170.24**
≤ 9 months	105.06	(265.40)	402.67	(261.65)	107.50	(152.93)	158.44	(251.71)					
> 9 months	153.96	(170.67)	99.17	(153.06)	15.45	(51.26)	98.03	(149.20)					
**Educational Services**	**9,250.30**	**(7,817.55)**	**9,743.14**	**(9,356.48)**	**17,752.05**	**(10,077.91)**	**12,082.50**	**(9,655.09)**	**5,330.24**	**(1,690.63)**	**6,752.26 (1117.46)**	**4562.08**	**8942.44**
≤ 9 months	9,253.24	(6,890.10)	11,628.04	(11,054.43)	18,886.95	(9,242.69)	13,072.46	(9,397.63)					
> 9 months	9,247.35	(8,878.56)	8,715.01	(8,699.15)	16,513.98	(11,235.64)	11,196.75	(9,920.14)					
**Lost productivity**	**16.88**	**(59.48)**	**-**		**82.61**	**(315.73)**	**33.42**	**(182.09)**	**12.97**	**(78.91)**	**20.45 (24.39)**	**-27.35**	**68.25**
≤ 9 months	15.00	(40.99)			33.33	(88.76)	18.82	(59.48)					
> 9 months	18.75	(75.00)			136.36	(452.27)	46.15	(243.71)					
**Other household**	**521.25**	**(2,040.87)**	**461.22**	**(912.33)**	**710.09**	**(1,289.46)**	**565.95**	**(1,583.72)**	**38.92**	**(236.73)**	**527.03 (153.73)**	**225.72**	**828.34**
≤ 9 months	816.00	(2,876.55)	902.00	(1,448.50)	675.00	(1,308.33)	781.41	(2,158.00)					
> 9 months	226.50	(384.80)	240.83	(416.75)	748.36	(1,331.16)	378.10	(793.94)					
**Total costs excluding Education**	**2,548.77**	**(4,914.31)**	**3,286.65**	**(5,160.29)**	**6,568.22**	**(8,890.53)**	**3,997.12**	**(6,633.79)**	**696.88**	**(674.54)**	**3,300.24 (644.21)**	**2037.62**	**4562.86**
≤ 9 months	3,536.99	(6,826.23)	6,237.77	(8,081.53)	4,491.30	(6,058.40)	4,350.41	(6,654.83)					
> 9 months	1,560.56	(1,109.08)	1,811.09	(2,083.45)	8,833.95	(11,076.71)	3,689.11	(6,686.81)					
**Total Costs Including Education**	**11,799.07**	**(10,186.17)**	**12,488.50**	**(13,179.43)**	**24,320.28**	**(16,381.95)**	**15,914.10**	**(14,167.56)**	**5,883.05**	**(2,075.50)**	**10,031.05 (1,822.27)**	**6459.47**	**13602.62**
≤ 9 months	12,790.23	(11,074.28)	17,865.81	(18,304.72)	23,378.26	(9,885.65)	17,422.87	(12,744.64)					
> 9 months	10,807.90	(9,470.89)	9,799.85	(9,616.75)	25,347.93	(21,924.54)	14,598.77	(15,344.21)					

PCHL = permanent childhood hearing loss ≥40 dB; HCG = Hearing Comparison Group; Diff = Difference; CI = confidence interval SD = standard deviation; SE = standard error.
